# The Effect of Nutrition Intervention With Oral Nutritional Supplements on Ovarian Cancer Patients Undergoing Chemotherapy

**DOI:** 10.3389/fnut.2021.685967

**Published:** 2021-06-25

**Authors:** Nan Qin, Guichun Jiang, Xu Zhang, Di Sun, Meishuo Liu

**Affiliations:** ^1^Department of Gynecology, Cancer Hospital of China Medical University, Liaoning Cancer Hospital & Institute, Shenyang, China; ^2^Department of Nursing, Cancer Hospital of China Medical University, Liaoning Cancer Hospital & Institute, Shenyang, China; ^3^School of Nursing, China Medical University, Shenyang, China; ^4^School of Nursing, Liaoning University of Traditional Chinese Medicine, Shenyang, China; ^5^School of Nursing, Norman Bethune Health Science Center of Jilin University, Changchun, China

**Keywords:** ovarian cancer, chemotherapy, oral nutritional supplement, nutritional status, patient-generated subjective global assessments

## Abstract

**Background:** Ovarian cancer is the third most common gynecological malignancy in the world and it is under a higher incidence of malnutrition. Chemotherapy is currently a common treatment for ovarian cancer, but the resulting side effects can exacerbate malnutrition. Our aim was to investigate the beneficial effects of oral nutrition supplements (ONS) on ovarian cancer patients undergoing chemotherapy.

**Methods:** Single-blinded randomized controlled trial. Patients with ovarian cancer receiving chemotherapy were randomly assigned either to the ONS or non-ONS groups via a simple randomization. The ONS group was given 250 mL ONS each time (1.06 kcal, 0.0356 g of protein per mL), three times a day, and nutrition education. Control group received nutrition education alone. The primary outcome was the nutritional risk of the patients as assessed by the Patient-Generated Subjective Global Assessment (PG-SGA). The secondary outcome was the results of the participants' biochemical tests at each measurement time point. Data were collected (T0) at baseline, (T1) post intervention at 3 weeks, (T2) 9-week follow-up, (T3) 15-week follow-up. Generalized estimating equation models were used to compare the changes in outcomes over time between groups.

**Results:** 60 participants (30 ONS, 30 controls) completed the trial, and data was analyzed. For baseline comparisons, no significant differences were found between the two groups. A progressive trend toward amelioration in PG-SGA scores over time was found within the ONS group, with scores decreasing from 9.27 ± 1.68 at baseline (T0) to 5.87 ± 2.06 after the intervention (T3). Furthermore, ONS group achieved a significantly greater reduction in PG-SGA score at the T1 (*p* = 0.03, confidence interval −2.23 to −0.11), T2 (*p* = 0.001, confidence interval −2.86 to −0.74) and T3 (*p* < 0.001, confidence interval −3.81 to −1.53), than the control group. In terms of biochemical test results, patients in the ONS group had better leukocytes, lymphocytes, Hemoglobin, Albumin and Total Protein than the control group at different time points, with statistical differences between the two groups (*p* < 0.05).

**Conclusions:** The present study demonstrated that ONS can significantly reduce the nutritional risk of patients undergoing chemotherapy for ovarian cancer. In addition, we also found that nutritional education seems to have a positive effect on reducing the nutritional risk of patients especially at the beginning of chemotherapy.

## Introduction

In the 21st century, cancer is considered to be the main cause of death and the most important obstacle to increasing life expectancy for people around the world. According to Global Cancer Report data published by the World Health Organization in 2020, the number of new cancer cases worldwide in 2020 is expected to be 19.29 million, including 10.06 million for men and 9.23 million for women; and the number of cancer deaths worldwide is expected to be 9.96 million (1). The global cancer burden has further increased. Ovarian cancer is the third most common gynecologic malignancy in the world (2). In 2020, more than 300,000 new cases of ovarian cancer are expected worldwide, with more than 190,000 deaths (3). The incidence and mortality of ovarian cancer in China are also increasing year by year, with approximately 52,100 new cases and about 22,500 deaths each year (4). Although its incidence is not as high as that of endometrial cancer, the lack of appropriate modality or biomarker for early screening and diagnosis of ovarian cancer makes it difficult to make effective predictions, and ultimately most patients diagnosed with ovarian cancer have advanced stage (nearly 75%) and therefore ovarian cancer has the highest mortality rate among gynecologic malignancies (3).

The nutritional status of ovarian cancer patients is not encouraging. Most patients with advanced ovarian cancer have peritoneal metastases, which are often manifested as abdominal pain and bloating, decreased appetite, nausea, and even intestinal obstruction that affect nutrient intake. Moreover, the Society of Gynecologic Oncology and the American Society of Clinical Oncology Clinical Practice Guideline recommend that the standard of treatment for advanced ovarian cancer remains primary cytoreductive surgery and chemotherapy (5). The trauma of surgery and the toxic effects of chemotherapy further exacerbate the malnutrition of ovarian cancer patients. Patient-Generated Subjective Global Assessment (PG-SGA) has been demonstrated to correlate with multiple clinical indicators of nutritional status and is most appropriate for identifying malnutrition in gynecologic cancer patients (6). We used the PG-SGA to assess the nutritional status of 201 Chinese patients undergoing postoperative chemotherapy for ovarian cancer and found that their nutritional status was generally poor, with 76.1% being moderately to severely malnourished and only 9.0% being well-nourished and not requiring nutritional intervention (7). Similar results have been reported from studies in other countries (8, 9). The nutritional status of cancer patients is the key to the patient's treatment outcome and survival time. Adequate and reasonable assessment of patients' nutritional risk to improve nutritional status and provide standardized nutritional support is of great clinical value to ameliorate the quality of life and prolong the survival time of cancer patients.

The European Society for Clinical Nutrition and Metabolism (ESPEN) and The Chinese Society for Parenteral and Enteral Nutrition (CSPEN) recommend oral nutritional supplement (ONS) provision during treatment to increase oral intake in malnourished cancer patients (10). ONS is an effective way of nutritional support, which can strengthen the nutrient content of protein, carbohydrate, fat, minerals and vitamins in food, and provide balanced nutrients to meet the body's demand for nutrients. Most of current research on the use of ONS has focused on esophageal cancer, gastrointestinal cancer, and head and neck cancer and has generally found that ONS can improve nutritional outcomes and better quality of life in cancer patients (11–13). However, few studies have reported the effect and improvement of ONS on the nutritional risk of ovarian cancer patients. Given the prevalence of malnutrition in ovarian cancer patients and the potential negative effects of malnutrition on cancer treatment, survival, and quality of life, research on the effect of ONS on ovarian cancer patients receiving chemotherapy is further required.

In the current study, we investigated the effects of ONS on ovarian cancer patients undergoing chemotherapy by examining changes in PG-SGA score at different time points.

## Materials and Methods

### Study Design

This study is a randomized controlled trial, which was conducted in China from May 2020 to October 2020. It was designed to investigate the effect of ONS on the nutritional risk and immune function of patients undergoing postoperative chemotherapy for ovarian cancer and to evaluate the clinical value of ONS.

The study complied with the Declaration of Helsinki and was approved by the Ethics Committee of Liaoning Cancer Hospital & Institute on October 17, 2018. The study was registered at http://www.chictr.org.cn (ChiCTR2100044785). Patients were provided written informed consent explaining all of the details when they joined the study, and informing them that their participation was entirely voluntary and they could withdraw from the study at any time. All patients who agreed to participate in the study were required to sign an informed consent form prior to the start of the intervention trial. The CONSORT statement was adopted for the study design and reporting.

### Study Participants

Patients were recruited from the gynecologic oncology department of the Liaoning Cancer Hospital & Institute in Shenyang, mainland China. Subjects were selected by initial identification in the electronic medical record based on patient treatment and identifying information. A second assessment was performed in the electronic medical record to explore whether they met the criteria for participation.

Subjects met the following inclusion criteria: (a) age ≥ 18 years old; (b) had a confirmed diagnosis of ovarian cancer and had completed primary treatment and decided to receive chemotherapy treatment; (c) were fluent in Mandarin Chinese. Exclusion criteria were: (a) past history of cognitive disorders, mental conditions or major sleep disorders; (b) already on a similar nutritional supplement; (c) severe dysphagia; (d) cancer metastasis.

### Randomization

In this prospective study, patients were randomly assigned either to the ONS or non-ONS groups via a simple randomization using the Statistical Analysis Software (SAS) 9.4 (SAS Institute Inc., Cary, North Carolina, U.S.). Participants, nurses, and researchers were not blinded because of practical impossibilities. Outcome assessors were blinded to the participants' group allocations during the data collection phases for purposes of examining differences between the groups. The laboratory technicians analyzing biochemistry parameters were also blinded to the group assignment.

### Sample Size

We used G^*^Power software to choose F tests (ANOVA: Repeated measures, between factors) to calculate the sample size. Given α = 0.05, power (1-β) = 0.80 and effect size *f* = 0.25, twenty-seven women were required to take part in each group after analyses of the sample size. Considering a potential attrition rate of 10%, sixty participants were recruited and randomly assigned to the intervention and control groups in a 1:1 ratio (*n* = 30 participants/group).

### Intervention

The research team members explained the purpose of the study and invited a professional nutritionist to educate ovarian cancer patients and their caregivers (relatives of the patients or contracted by them) about nutrition and health knowledge. Nutritional health education content mainly includes: displaying photos of nutritious foods, designing healthy recipes and paying attention to nutritional matching, and providing prevention and solutions to common nutritional problems of patients with ovarian cancer. In addition, patients assigned to the ONS group were required to receive 250 ml of oral nutritional supplements (Ensure®, 1.06 kcal, 0.0356 g of protein per mL, Abbott Laboratories USA) three times a day. In order to improve compliance with oral nutritional supplements, members of the research team used smartphones to provide daily reminders to patients in the ONS group via WeChat software. The possible side effects of ONS treatment will also be monitored, and the trial will interrupt or reduce the dose of oral nutrients when patients experience symptoms such as diarrhea, vomiting, and abdominal pain.

### Controls

Except not receiving ONS treatment, procedures for control group participants were identical to those for the intervention group following randomization. Participants' nutritional questions during the follow-up period are collected and answered online by a nurse. Moreover, ONS treatment would also be initiated in the control group patients when a further weight loss ≥ 5% occurred within 1 month after discharge from the hospital (14).

### Data Collection

The nutritional assessment was performed using the PG-SGA, with the patient self-assessment part being completed by themselves and the remaining part being assessed in detail by a nurse certified in nutritional care who was blinded to the grouping of participants. The participants were assessed four times: (T0) at baseline, (T1) post-intervention at 3 weeks, (T2) 9-week follow-up, (T3) 15-week follow-up. The assessments took place when the patients returned to the hospital for their hospitalized chemotherapy and results were recorded at each time point.

### Baseline Variables

Eligible patients who signed an informed consent form were invited to participate in the baseline measurements. The research team developed a socio-demographic information questionnaire that included questions about the participant age, marital status, education level, living arrangement and primary caregiver. Each question in the questionnaire had preset options, which were selected by the patients themselves according to their actual situation. The patient's medical history information was derived from their respective electronic medical records, including time since diagnosis, recurrence, combined chronic diseases and cancer stage.

### Primary Outcome Measures

The PG-SGA was developed by FD Ottery, and it was used as a prognostic tool to assess the nutritional status of cancer patients (15). PG-SGA is widely used in various countries and studies have confirmed its high sensitivity to changes in nutritional status and risk over time (16–18). The Chinese version of the PG-SGA used in this study was translated by experts at the Chinese Society for Oncological Nutrition & Supportive Care and is the only nutritional assessment tool recommended by The China Anti-Cancer Association that is applicable to cancer patients (19). It included questions related to nutritional symptoms, short-term weight loss, and other medical history. Referring to the operational criteria developed by the American Academy of Nutrition and Dietetics, we used the PG-SGA to assess the nutritional risk of patients with ovarian cancer. A higher PG-SGA score indicates a poorer risk status.

### Secondary Outcome Measures

In the case of malnutrition, the leukocytes of cancer patients will decrease significantly under the effect of chemotherapeutic drugs, resulting in the inability of patients to complete their chemotherapy plans, thus affecting the therapeutic effect. In addition, when the level of plasma protein is low, the absorption, distribution, metabolism and excretion of chemotherapy drugs will also be impaired, leading to an increase in adverse reactions to chemotherapy and a decrease in the body's ability to tolerate chemotherapy. Therefore, the secondary outcome indicator was the participant's biochemical test at each measurement time point, which was obtained from the patient's electronic medical record at the hospital. The nutrition-related biochemical indicators collected were: leukocytes, lymphocytes, red blood cells, hemoglobin, albumin, and total protein.

### Statistical Analysis

The results were analyzed using SPSS Statistics version 26.0 (IBM Corp., Armonk, NY, USA). Statistical significance was considered at *P* = 0.05. Two independent sample *t*-tests and chi-square tests or Fisher's exact tests were used to assess baseline data, such as demographic, and biochemical test data, as well as differences between experimental and comparison groups. Generalized estimating equation (GEE) models with the appropriate link function and distribution assumptions were used to compare the differential changes of the outcomes across time and between groups (20). A feature of longitudinal data is that repeated measurements are performed on individuals in chronological order, so the inter-individual data obtained are generally non-independent. The GEE model solves the problem of the correlation of the response variables in the longitudinal data, and the parameter estimates can be obtained through the marginal model (21).

## Results

**Figure 1:**
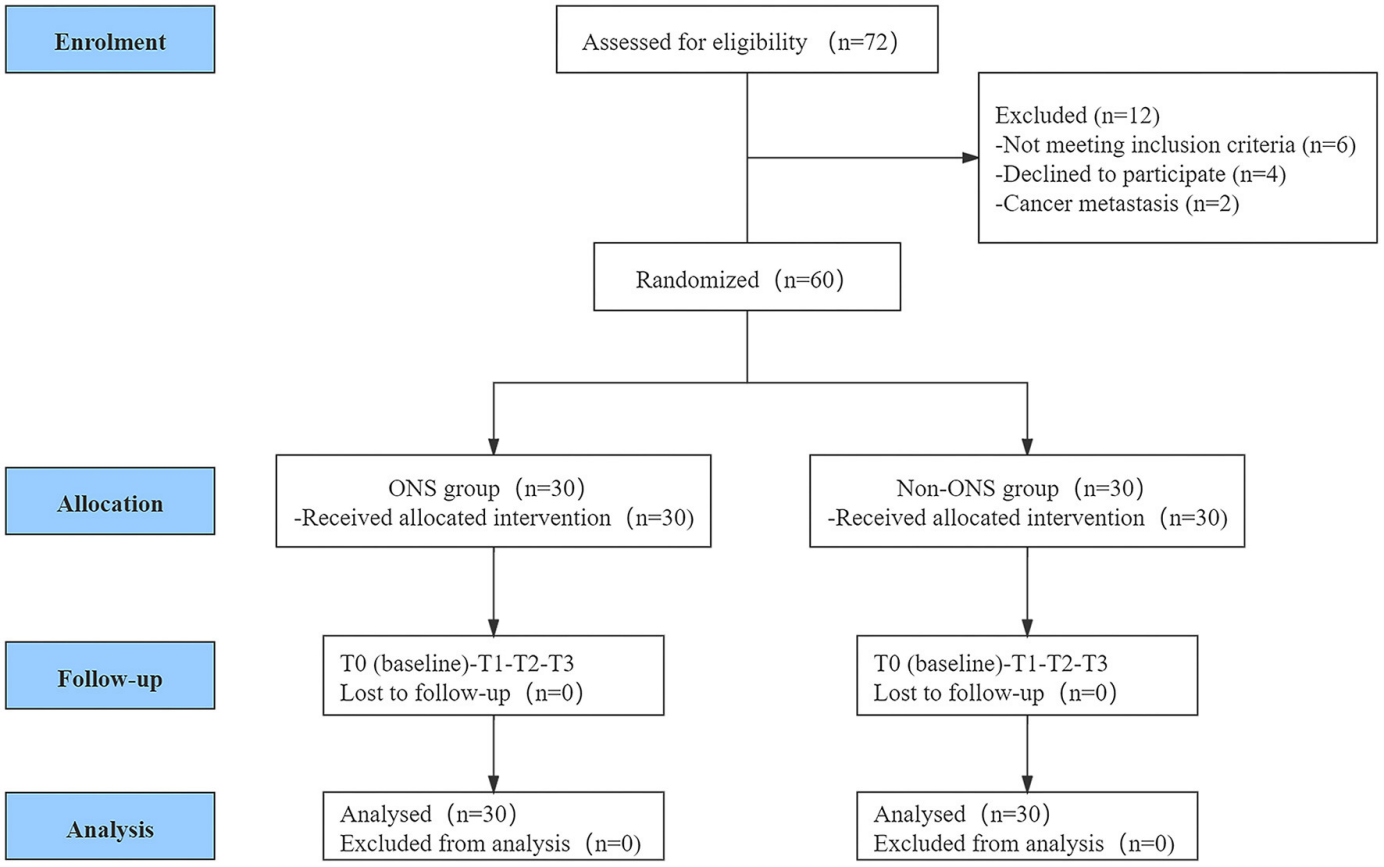
CONSORT flow diagram.

A total of 72 women from the gynecologic oncology department at Liaoning Cancer Hospital & Institute were screened for eligibility. Among them, 60 women met the inclusion criteria and were randomly assigned into the ONS group (*n* = 30) and control group (*n* = 30). The CONSORT flow diagram of the participants through the trial is shown in Figure 1.

Tables 1, 2 show the differences in the sociodemographic information and biochemical indicators of the participants at the baseline time point. The two groups were well-balance and no statistical difference was found.

**Table 1:** Socio-demographic characteristies of study participants.

**Socio demographic characteristics**		**ONS group (*N* = 30)**	**Control group (*N* = 30)**	***t*/χ^2^**	***P*-value**
Age (Mean ± SD)		53.33 ± 10.32	54.67 ± 11.91	−0.46	0.65
PG-SGA scores		9.27 ± 1.68	9.83 ± 2.09	−1.16	0.25
Marital status	Married	28	29	1.194^a^	1.00^a^
	Unmarried	1	0		
	Divorced	1	1		
Education level	Elementary school or below	11	9	1.59^a^	0.46^a^
	Junior high school	16	20		
	High school or college	3	1		
Living arrangement	Alone	0	1	2.31^a^	0.64^a^
	With spouse	22	24		
	With children	7	5		
	With parents	1	0		
Primary caregiver	Spouse	17	15	6.13^a^	0.09^a^
	Parents	1	0		
	Children	12	10		
	Relatives or Others	0	5		
Combined chronic diseases	Yes	18	22	1.20	0.27
	No	12	8		
Time since diagnosis	3–12 months	30	27	1.40^b^	0.24^b^
	>12 months	0	3		
Stage	I	2	3	0.64^a^	0.93^a^
	II	3	3		
	III	22	20		
	IV	3	4		
Recurrence	Yes	1	2	0.00^b^	1.00^b^
	No	29	28		

*Combined chronic diseases include chronic atrophic gastritis, diabetes, hypertension, coronary heart disease, chronic obstructive pulmonary disease*.

a *Fisher's Exact Test*.

b *Continuity Correction*.

*PG-SGA, Patient-Generated Subjective Global Assessment*.

**Table 2:** Baseline characteristics of the biochemical indicators of the study participants.

		**ONS group (*N* = 30) (Mean ± SD)**	**Control group (*N* = 30) (Mean ± SD)**	***t***	***P*-value**
Leukocytes	10^9^/L	6.61 ± 2.63	6.11 ± 1.79	0.88	0.39
Lymphocytes	10^9^/L	1.54 ± 0.71	1.51 ± 0.88	0.14	0.89
Red blood cells	10^12^/L	3.96 ± 0.47	4.08 ± 0.52	−0.97	0.34
Hemoglobin	g/L	115.23 ± 10.65	118.87 ± 18.26	−0.94	0.35
Albumin	g/L	39.37 ± 5.87	40.41 ± 6.28	−0.66	0.51
Total protein	g/L	70.20 ± 7.93	69.75 ± 9.93	0.19	0.85

### Adherence to the Intervention

During the study period, all patients in the ONS group reported that they completed the intervention goal of 750 ml of oral nutritional supplements every day without serious adverse events and both groups of patients had relatively high adherence to their assigned treatment.

### Primary Outcome

**Figure 2:**
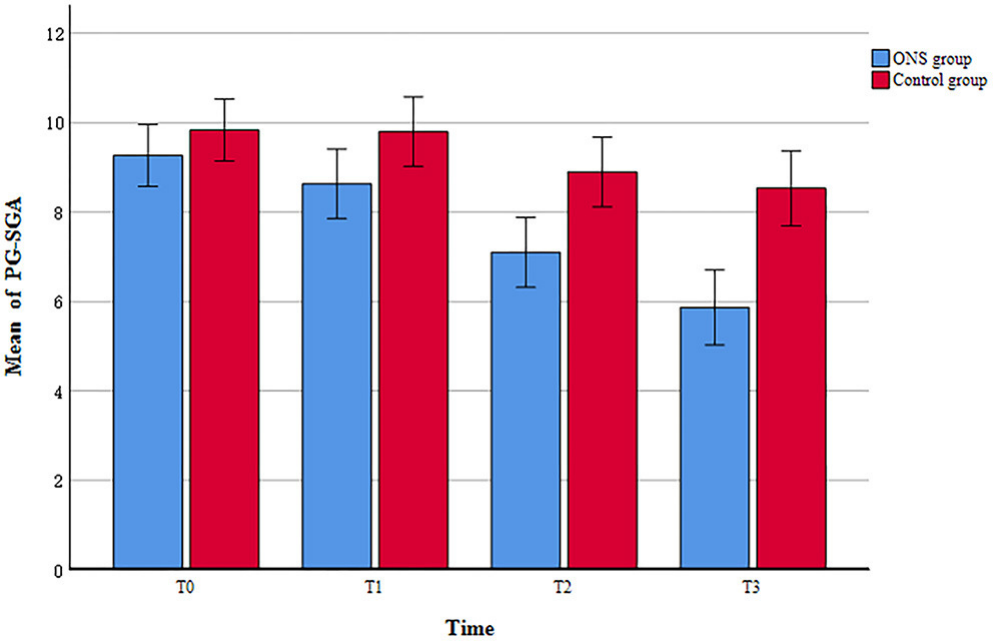
The change of patient-generated subjective global assessment (PG-SGA) scores between ONS and control groups.

The PG-SGA score were used to evaluate the changes in nutritional risk between the ONS group and the control group at each time point. The examination of nutritional risk changes across time indicated that the ONS group achieved a significantly greater reduction in PG-SGA score at the T1, T2, and T3, than the control group (*p* < 0.05; Table 3). In addition, a progressive trend toward improvement in PG-SGA scores over time was found within the ONS group, with scores decreasing from 9.27 ± 1.68 at baseline (T0) to 5.87 ± 2.06 after the intervention (T3) (Figure 2). On the other hand, the PG-SGA score of the control group also improved slightly, but there was only T1 compared with T2 and T3 had a statistical difference, and T2 compared with T3 did not change significantly (Supplementary Table 1).

**Table 3:** GEE model estimates of the differences in changes from baseline (mean change with confidence intervals) in nutritional risk and biomarkers between groups across time (*n* = 60).

	**Time**	**ONS-C estimated mean difference (95% CI)**	***P*-value**
PG-SGA	T1	−1.17 (−2.23, −0.11)	**0.03**
	T2	−1.80 (−2.86, −0.74)	**0.001**
	T3	−2.67 (−3.81, −1.53)	** <0.001**
Leukocytes	T1	−0.35 (−1.69, 1.00)	0.61
	T2	−0.05 (−0.95, 0.85)	0.92
	T3	1.33 (0.53, 2.13)	**0.01**
Lymphocytes	T1	0.41 (−0.04, 0.86)	0.07
	T2	0.24 (−0.12, 0.60)	0.19
	T3	0.36 (0.04, 0.68)	**0.03**
Red blood cells	T1	0.05 (−0.20, 0.30)	0.69
	T2	0.12 (−0.13, 0.36)	0.36
	T3	0.02 (−0.23, 0.28)	0.85
Hemoglobin	T1	1.83 (−4.48, 8.15)	0.57
	T2	8.87 (2.78, 14.95)	**0.004**
	T3	11.77 (6.94, 16.59)	** <0.001**
Albumin	T1	3.71 (0.75, 6.68)	**0.01**
	T2	3.96 (2.04, 5.88)	** <0.01**
	T3	1.95 (−0.15, 4.05)	0.07
Total protein	T1	5.49 (−0.36, 11.34)	0.07
	T2	5.65 (2.58, 8.71)	** <0.01**
	T3	4.40 (1.90, 6.90)	** <0.01**

*T1, post-intervention at 3 weeks; T2, 9-week follow-up; T3, 15-week follow-up. ONS, oral nutritional supplement; C, Control*.

*PG-SGA, Patient-Generated Subjective Global Assessment; GEE, generalized estimating equations; CI, confidence interval. Bold values indicate of p < 0.05*.

### Secondary Outcomes

The results of the biochemical tests at each time point showed a gradual decrease in leukocytes and Lymphocytes with chemotherapy in both groups, but the ONS group had better leukocyte and Lymphocytes counts than the control group at the T3 (*p* = 0.01 CI 0.53–2.13; *p* = 0.03 CI 0.04–0.68). The hemoglobin and total protein of patients in the ONS group was statistically superior to the control group at T2 (*p* = 0.004 CI 2.78–14.95; *p* < 0.01 CI 2.58–8.71) and T3 (*p* < 0.001 CI 6.94–16.59; *p* < 0.01 CI 1.90–6.90). There was a significant improvement in the albumin of patients in the ONS group compared to the control group at T1 (*p* = 0.01 CI 0.75–6.68) and T2 (*p* < 0.01 CI 2.04–5.88), but there was no significant difference between the two groups at T3. For other biochemical indicator related to nutritional risk, red blood cells, no significant differences in the changes over time were observed across the both groups. All the above results are shown in Table 3.

## Discussion

The human body cannot maintain normal physiological, biochemical and immune functions, growth and development, metabolism, repair and other vital activities without nutritional support. However, cancer patients are more vulnerable to malnutrition due to the metabolic changes caused by the disease and the side effects of treatment. Some studies have shown that 40–80% of cancer patients have malnutrition, 20% die from malnutrition, 50–80% have cachexia, and 30% die directly from cachexia (15, 22). Malnutrition has also been proven to be one of the important causes of death in patients with ovarian cancer (8, 23). Malnutrition seriously weakens the effectiveness of treatment, leading to increased complications, lower quality of life, longer hospital stays, increased medical costs, and shorter survival times (24). Cancer patients undergoing chemotherapy need adequate nutritional interventions to maintain weight for a better prognosis, because chemotherapy increases their risk of malnutrition (16). In this study, a 15-week follow-up investigation of the intervention and control groups was conducted by designing a randomized controlled trial, and it was found that the nutritional risk (PG-SGA score) of patients in the ONS group was significantly improved. In terms of biochemical parameters, patients in the ONS group also had better overall Leukocytes, Lymphocytes, Hemoglobin, Albumin and Total Protein than the control group. These findings indicate that ONS can reduce the nutritional risk of patients with ovarian cancer and has clinical application value.

ONS is an important intervention of effective, non-invasive nutritional support for patients who are able to consume some food but whose intake is insufficient to meet their full nutritional needs. The present study found that ONS can reduce the nutritional risk of patients with ovarian cancer regardless of whether it is compared with the control group or at various time points in the ONS group. Similar results have been reported in other studies (13, 14). In addition to the significant improvement in nutritional risk identified in ovarian cancer chemotherapy patients receiving ONS in this study, a slight improvement in nutritional risk was also found in control patients not receiving ONS at the beginning of chemotherapy, which may be attributed to the nutrition education provided to patients and their caregivers throughout this study. Nutrition education has been shown to reduce the incidence of malnutrition in oncology patients and side effects such as nausea and vomiting caused by chemotherapy (25, 26). Many patients and their caregivers aggravate the occurrence of malnutrition due to wrong dietary behaviors, which may eventually lead to interruption of treatment due to nutritional problems. Cong et al. surveyed 535 participants from 18 hospitals in China about dietary knowledge and behavior, they found that 70% of the patients had no clear idea of what was a scientific diet and 99.6% of patients have made mistakes about dietary knowledge (27). Therefore, it is an urgent and far-reaching task for clinical health care professionals to improve the nutritional risk of most oncology patients by properly guiding their dietary behavior and consuming adequate structured nutrition via oral intake. However, the current nutrition education for cancer patients has not yet formed an evidence-based systematic practice program. It is suggested that future research can conduct in-depth discussions in terms of the timing, frequency, and the selection of educational objects for nutrition education.

Chemotherapy can both fundamentally improve malnutrition in cancer patients through its anti-cancer effects, but may also cause or aggravate malnutrition in patients due to its adverse effects. 30–90% of cancer patients have cancer chemotherapy-related anemia (CRA), which is mainly manifested as a decrease in the number of red blood cells per unit volume in the peripheral blood, a decrease in hemoglobin concentration, or a decrease in hematocrit below the normal level (28, 29). According to the literature, hemoglobin concentration differed by tumor site and was lowest in patients with ovarian cancer, suggesting that patients with gynecologic cancers may be more prone to CRA (30). CRA can cause multiple organ ischemia-hypoxic changes and decreased immunity in patients, aggravate disease progression, affect prognosis, and seriously affect the quality of life of patients (31). Chemotherapy may also lead to weakened body functions in cancer patients, which will affect the ability of albumin synthesis. Albumin is a major component of human plasma proteins, which maintains important physiological functions such as plasma colloid osmotic pressure and transport, and it also improves the tolerance of enteral feeding (32). Li et al. evaluated the correlation between albumin and survival time before radiotherapy and chemotherapy in 512 cases of nasopharyngeal carcinoma, and found that the albumin level was correlated with the patient's overall survival time and was an independent prognostic factor for overall survival time (33). Another study found that low levels of hemoglobin, albumin, and lymphocytes may be an important risk factor for recurrence-free survival and overall survival in postoperative cancer patients (34). Therefore, it is particularly important to closely monitor the biochemical indicators of cancer patients during chemotherapy, which have become an important reference for evaluating nutritional risk and whether to continue chemotherapy. In the present study, although most of the biochemical indicators were within the normal range at all time points, the improvement of biochemical indicators in the ONS group was still superior to that in the control group, and the findings highlight even more the importance of ONS in chemotherapy. However, contrary to our findings, previous studies found that ONS intervention did not have a positive effect on biochemical indicators (16, 35). This may be due to the fact that ovarian cancer patients in the ONS group in our study were more compliant with the intervention and received comprehensive and timely nutrition education.

The present study also has some limitations. First, while this study concluded that ONS had a positive effect on the nutritional risk of ovarian cancer patients, the influence of a single study center and a small sample size may have biased the findings, although patient compliance was good in this study. Secondly, this study could not be double-blinded due to practical objective factors, which may have contaminated participants between different groups. Finally, while the use of the PG-SGA in this study was conducted in full accordance with the operational standards recommended by the American Academy of Nutrition and Dietetics, linguistic validity of the Chinese version of the PG-SGA remains to be confirmed.

## Conclusion

We can conclude that ONS can significantly improve the nutritional risk of patients undergoing chemotherapy for ovarian cancer compared to the control group and that ONS has the advantages of being simple, easy to administer and physiologically appropriate and should be the first choice for enteral nutrition therapy. Moreover, the nutritional risk of the control group, which received only nutrition education, also showed a slight improvement, especially at the beginning of chemotherapy. This emphasizes the importance of nutritional education in the nutritional support therapy of cancer patients.

## Data Availability Statement

The raw data supporting the conclusions of this article will be made available by the authors, without undue reservation.

## Ethics Statement

The study complied with the Declaration of Helsinki and was approved by the Ethics Committee of Liaoning Cancer Hospital & Institute on October 17, 2018. The study was registered at http://www.chictr.org.cn (ChiCTR2100044785).

## Author Contributions

NQ and GJ designed the study. NQ was the main author of the manuscript, performed all the assessments of nutritional risk, analyzed, and interpreted data. NQ, XZ, DS, and ML supervised the project and assisted with the statistical analysis, writing the manuscript, and interpretation of the results. NQ and GJ assisted in interpretation of the results and writing the manuscript. All authors contributed to the article and approved the submitted version.

## Conflict of Interest

The authors declare that the research was conducted in the absence of any commercial or financial relationships that could be construed as a potential conflict of interest.
