# Supplementary material for: The Effect of Nutrition Intervention With Oral Nutritional Supplements on Ovarian Cancer Patients Undergoing Chemotherapy
Source: Front Nutr. 2021 Jun 25;8:685967. doi: 10.3389/fnut.2021.685967 (PMC8267173; doi:10.3389/fnut.2021.685967)
Supplement: Supplementary file 1 [file Table_1.DOCX]

Table S1

GEE model estimates of the differences in changes from baseline（mean change with confidence intervals）of the respective nutritional risk and biomarkers of the two groups across time (n = 60).

|  | Time | | ONS group  (n=30) |  | Control group  (n=30) |  |
| --- | --- | --- | --- | --- | --- | --- |
|  |  | | MD (95%CI) | P-Value | MD (95%CI) | P-Value |
| PG-SGA | T1 | -T2 | 1.53(1.09,1.97) | **<0.001** | 0.90(0.52,1.28) | **<0.001** |
|  |  | -T3 | 2.77(2.28,3.25) | **<0.001** | 1.27(0.81,1.73) | **<0.001** |
|  | T2 | -T3 | 1.23(0.87,1.60) | **<0.001** | 0.37(-0.15,0.89) | 0.17 |
| Leukocytes | T1 | -T2 | 0.20(-0.99,1.39) | 0.74 | 0.50(-0.24,1.24) | 0.18 |
|  |  | -T3 | 0.41(-0.83,1.64) | 0.52 | 2.08(1.33,2.84) | **<0.001** |
|  | T2 | -T3 | 0.20(-0.22,0.63) | 0.35 | 1.58(1.03,2.14) | **<0.001** |
| Lymphocytes | T1 | -T2 | 0.02(-0.14,0.18) | 0.81 | -0.15(-0.41,0.11) | 0.25 |
|  |  | -T3 | -0.02(-0.23,0.20) | 0.87 | -0.07(-0.29,0.15) | 0.55 |
|  | T2 | -T3 | -0.04(-0.22,0.14) | 0.68 | 0.08(-0.06,0.23) | 0.27 |
| Red Blood Cells | T1 | -T2 | 0.07(-0.04,0.17) | 0.20 | 0.13(-0.09,0.35) | 0.25 |
|  |  | -T3 | 0.22(0.04,0.40) | **0.02** | 0.19(-0.01,0.39) | 0.06 |
|  | T2 | -T3 | 0.15(0.01,0.29) | **0.03** | 0.06(-0.10,0.22) | 0.45 |
| Hemoglobin | T1 | -T2 | -3.47(-5.87,-1.07) | **0.005** | 3.57(-2.88,10.02) | 0.28 |
|  |  | -T3 | -8.77(-11.62,-5.91) | **＜0.001** | 1.17(-4.98,7.32) | 0.71 |
|  | T2 | -T3 | -5.30(-7.74,-2.86) | **＜0.001** | -2.40(-6.40,1.60) | 0.24 |
| Albumin | T1 | -T2 | -2.92(-4.26,-1.57) | **<0.01** | -2.67(-5.27,-0.06) | **0.04** |
|  |  | -T3 | -4.18(-5.50,-2.85) | **<0.01** | -5.94(-9.10,-2.77) | **<0.01** |
|  | T2 | -T3 | -1.26(-2.12,-0.41) | **<0.01** | -3.27(-5.35,-1.20) | **<0.01** |
| Total Protein | T1 | -T2 | -5.07(-9.40,-0.74) | **0.02** | -4.92(-8.85,-0.99) | **0.01** |
|  |  | -T3 | -7.16(-11.66,-2.66) | **<0.01** | -8.25(-12.26,-4.24) | **<0.01** |
|  | T2 | -T3 | -2.09(-3.05,-1.12) | **<0.01** | -3.33(-5.92,-0.75) | **0.01** |

Notes: T1, post-intervention at 3 weeks; T2, 9-week follow-up; T3, 15-week follow-up. ONS= oral nutritional supplement;

Abbreviations: PG-SGA, Patient-Generated Subjective Global Assessment. GEE, generalized estimating equations; MD, Estimated Mean Difference; CI, confidence interval.
